# 

**Published:** 1877-02

**Authors:** 


### CONTENTS.

COMMUNICATIONS

Concerning the Causes of Failures in the use of Cohesive Foil, the Mallet, etc., . . . 53
Gold Foil, . . . . . . . . . . 62
Exposed Pulps, and how Nature Cures Them, . 69
A Strange Case. Is There a Parallel? . . . 73
Principles Involved in the Construction of Artificial Teeth, . . . . . . . . 83

SELECTIONS.

The American Leech Trade, . . . . . 87
Tooth Ache Remedy, . . . . . . . 88
Salicylic Acid Mixtures, . . . . . . 88
To Make Leeches Bite, . . . . . . . 89

EDITORIAL.

"Fiat Justitia" Again, . . . . . . . 90
Mississippi Valley Dental Association, . . 91
To the Dental Profession, . . . . . . 92
Celluloid. What is it? . . . . . . 92

